# P-2344. Genomic Analysis of Monkeypox Virus (MPVX) Outbreak in Bangkok, Thailand in 2022-2023

**DOI:** 10.1093/ofid/ofae631.2496

**Published:** 2025-01-29

**Authors:** Opass Putcharoen, Khwankamon Rattanatumhi, Ananporn Supataragul, Pakamas Sangsub, Supaporn Wacharapluesadee

**Affiliations:** Division of Infectious Disease, Department of Medicine, Faculty of Medicine, Chulalongkorn University, Krungthep, Krung Thep, Thailand; Thai Red Cross Emerging Infectious Diseases Clinical Center, Pathum Wan, Krung Thep, Thailand; Thai Red Cross Emerging Infectious Diseases Clinical Center, Pathum Wan, Krung Thep, Thailand; Thai Red Cross Emerging Infectius Diseases Clinical Center, King Chulalongkorn memorial hospital, Bangkok, Krung Thep, Thailand; Emerging infectious diseases clinical center, King Chulalongkorn Memorial Hospital, Pathumwan, Krung Thep, Thailand

## Abstract

**Background:**

Since early May 2022, cases of mpox (monkeypox) have been reported in countries where the disease is not endemic, alongside continued reports from endemic regions. This marks the first time that multiple mpox cases and clusters have been concurrently reported in both non-endemic and endemic countries across diverse geographical areas. As of March 8, 2024, there were 94,776 confirmed mpox cases globally, with 721 cases in Thailand—most of which were reported from Bangkok.

Figure 1

**Figure:**
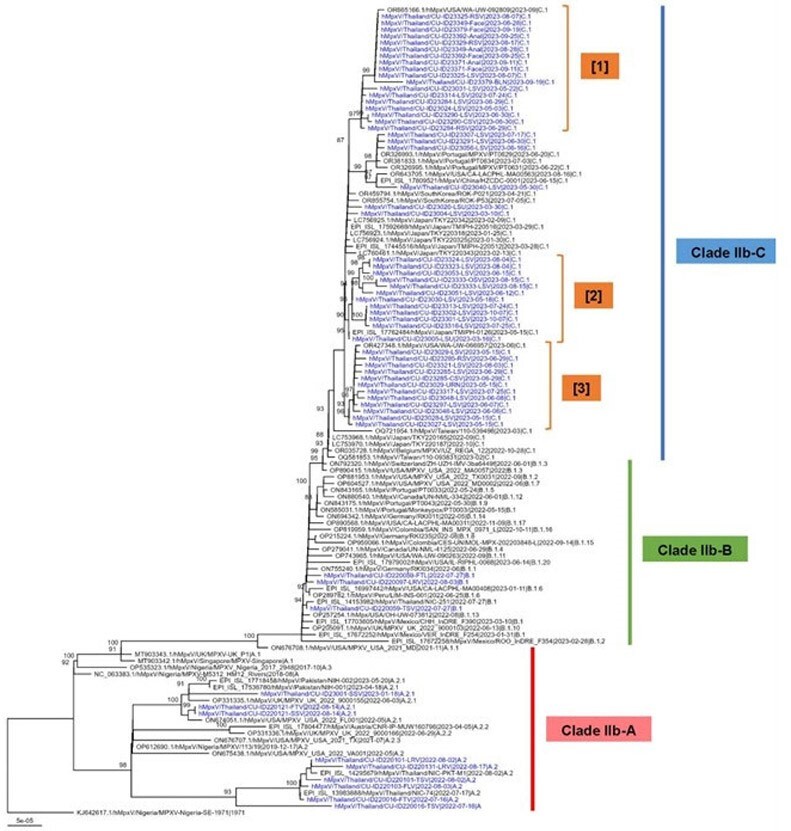

The phylogenetic tree was constructed from 60 Mpox genomes collected in Bangkok, Thailand, during 2022-2023. The tree was produced using the maximum likelihood method in IQ-TREE2 with 1000 replicates of ultrafast bootstrap and HKY+F+I nucleotide substitution model and visualized by ggtree library in R.

**Methods:**

We collected biological samples, mostly lesion swabs from suspected Mpox cases at STI Clinics in Bangkok, Thailand, from July 2022 to September 2023. Whole Genome Sequencing (WGS) was performed on 60 specimens from mpox cases in Bangkok, primarily lesion swabs, that were PCR-positive for MPXV with a cycle threshold (Ct) value below 30. A hybridization probe-capture–based approach was utilized for sequencing, specifically Illumina RNA Prep with Enrichment kit with the Viral Surveillance Panel. Consensus genomes were generated using an in-house pipeline based on reference mapping (BWA v0.7.17) to the Mpox reference genome from Nigeria (GenBank accession no. NC_063383.

**Results:**

Phylogenetic analysis from WGS revealed that the predominant MPXV clade in Bangkok is IIb, with most specimens belonging to lineage IIb-C (48/60), followed by IIb-A (9/60) and IIb-B (3/60) as shown in Figure 1. Lineage IIb-A was detected in samples from 2022, while IIb-B and IIb-C were prominent in 2023. We identified multiple clusters with similar genomes in lineage IIb-C from different individuals, with percent identity ranging from 99.970% to 99.999% (197,184 nucleotides), suggesting ongoing community transmission. There is no clear introduction of lineage IIb-C into Thailand, however, the phylogenetic tree indicates the transmission of locally transmitted lineage to other countries. We detected the unique activity of APOBEC mRNA editing in serial samples from immunocompromised hosts. No mutations associated with resistance to tecovirimat were identified.

**Conclusion:**

Our study demonstrated the introduction of MPXV from multiple countries, confirming ongoing community transmission in Bangkok during 2022-2023. Continuing genomic surveillance is crucial for tracking the epidemiology and viral evolution.

**Disclosures:**

All Authors: No reported disclosures

